# *FUSION*: a family-level integration approach for robust differential analysis of small non-coding RNAs

**DOI:** 10.1093/bioinformatics/btaf526

**Published:** 2025-09-18

**Authors:** Hukam C Rawal, Qi Chen, Tong Zhou

**Affiliations:** Department of Physiology and Cell Biology, University of Nevada, Reno School of Medicine, Reno, NV 89557, United States; Molecular Medicine Program, Department of Human Genetics, University of Utah School of Medicine, Salt Lake City, UT 84132, United States; Division of Urology, Department of Surgery, University of Utah School of Medicine, Salt Lake City, UT 84132, United States; Department of Physiology and Cell Biology, University of Nevada, Reno School of Medicine, Reno, NV 89557, United States

## Abstract

**Motivation:**

Beyond well-studied microRNAs, noncanonical small non-coding RNAs (sncRNAs) derived from longer parental templates such as tRNAs, rRNAs, and Y RNAs, are emerging as important regulators in various biological processes and diseases. Yet, analyzing these noncanonical sncRNAs from sequencing data remains challenging due to the intrinsic sequence heterogeneity and highly noisy nature. Conventional strategies either sum up all sequencing reads mapped to a parental RNA, which sacrifices the resolution of single sncRNA species, or treat each unique RNA species/sequence independently, which faces substantial noise in low-replicate settings.

**Results:**

Here, we introduce *FUSION* (Family-level Unique Small RNA Integration), a computational tool bridging these conventional approaches by first quantifying unique sncRNA species and then aggregating them into their respective parental RNA families. This family-level integration captures the contributions of individual sncRNA species while enhancing statistical power and robustness for differential abundance analysis. *FUSION* includes two modules: *FUSION_ms*, which reduces noise and amplifies signals for multiple-sample comparison to detect family-level abundance changes even with a small sample size, and *FUSION_ps*, which is powered by paired-sample analysis and optimized for “1-on-1” differential abundance analysis in single-case studies. Both modules are validated by cross-lab discoveries of dysregulated sncRNA families that could not be identified using conventional methods. In summary, *FUSION* provides a powerful framework for sncRNA sequencing data analysis, enhancing data interpretation and supporting small sample research.

**Availability and implementation:**

*FUSION* is available at https://github.com/cozyrna/FUSION and archived at https://doi.org/10.5281/zenodo.16929712.

## 1 Introduction

Small non-coding RNAs (sncRNAs) are found across all domains of life, including bacteria, archaea, and eukarya, and are involved in fundamental biological processes and diseases (Shi *et al.* 2022, Chen and Zhou 2023). Beyond the well-studied microRNAs (miRNAs), the sncRNA universe includes previously underexplored types that are derived from longer structured RNAs, such as transfer RNAs (tRNAs), ribosomal RNAs (rRNAs), Y RNAs (yRNAs), small nuclear RNAs (snRNAs), small nucleolar RNAs (snoRNAs), and vault RNAs (vtRNAs). This renewed wave of sncRNA discovery was especially augmented by the recently developed sncRNA sequencing (sncRNA-seq) tools like PANDORA-seq (Shi *et al.* 2021) and others (Shi *et al.* 2022, Tosar *et al.* 2024), which overcome the detection challenges posed by RNA modifications. These tools reveal that miRNAs account for only 0.1%–5% of total sncRNAs across diverse cells and tissues, while tRNA-derived small RNAs (tsRNAs, also known as tRNA fragments or tRFs) and rRNA-derived small RNAs (rsRNAs, also known as rRNA fragments or rRFs) dominate in many contexts (Shi *et al.* 2022, Chen and Zhou 2023). Given the heterogeneous sources of these noncanonical sncRNAs, we have previously developed the *SPORTS* bioinformatic pipeline (Shi *et al.* 2018) to systematically profile them, achieving superior performance in benchmarking tests (Di Bella *et al.* 2020).

However, our expanded capacity to discover and annotate heterogeneous sncRNAs (e.g. miRNA, tsRNA, rsRNA, and ysRNAs) has brought up new analytical challenges (Shi *et al.* 2022). A key question is how to interpret abundance changes between conditions, particularly how to leverage diverse sncRNA subtypes (e.g. unique tsRNA species/sequences from a parental tRNA template) to boost statistical power and reduce multiple testing burdens while maintaining biological relevance (e.g. different tsRNAs from the same parental tRNA may share similar functions despite length variations). Currently, two main conventional strategies are used, with tsRNAs as an example.

- Strategy I: Sums all reads mapped to a parental tRNA (e.g. genomic tRNA-Gly-GCC) into a single count (e.g. 800 reads in control versus 1000 reads in treated sample) for differential abundance analysis (Manning *et al.* 2025). This method is simple in categorizing and maintaining the biological origin of tsRNAs but overlooks the variation within the individual sncRNA species from the same parental RNA.
- Strategy II: Treats each unique tsRNA sequence (e.g. genomic 5′-tsRNA-Gly-GCC-31nt and genomic 5′-tsRNA-Gly-GCC-32nt) as an individual entity for statistical/bioinformatic analysis (Liu *et al.* 2023a, Hernandez *et al.* 2024). This approach is detailed for sequence identity but often suffers from low read counts and high variability of individual sncRNA species/sequences.

To balance and combine the strengths of both strategies, we propose a computational framework *FUSION* (Family-level Unique Small RNA Integration), which first quantifies unique sncRNA species (like Strategy II) and then statistically aggregates them into parental RNA families (e.g. genomic tsRNA-Gly-GCC) instead of simply summing up the sequencing reads as described in Strategy I. This family-level integration captures the contributions of individual sncRNA species while enhancing statistical power and robustness, especially in the research settings with small sample sizes. There are two modules in *FUSION: FUSION_ms* for multiple-sample comparison to detect family-level abundance changes between groups, and *FUSION_ps* for paired-sample analysis, which is optimized for “1-on-1” differential abundance analysis in single-case studies. Testing across multiple sncRNA-seq datasets of different disease conditions demonstrates that both modules can efficiently capture data signals and thus enhance statistical power. Importantly, both modules robustly identify common disease-induced sncRNA changes across independent datasets, which could not be achieved by the conventional methods (e.g. Strategy I and II). In summary, *FUSION* represents a powerful and versatile computational framework for sncRNA-seq data analysis, with the potential to redefine the interpretation of existing datasets and empower researchers facing the constraints of small sample size.

## 2 Materials and methods

### 2.1 *FUSION_ms* module

The *FUSION_ms* module is designed to identify differentially abundant sncRNA families between groups or correlated with continuous phenotype, where multiple samples exist. We first assume there are *m* samples (*s*_1_, *s*_2_, …, *s_m_*) in a sequencing dataset, and, for a given sncRNA family, there are *n* sncRNA species derived from this family, and there is only one direction of dysregulation among these sncRNA species. For each sncRNA species *i* that is derived from this given sncRNA family, we collect the log_10_-transformed abundance across all the samples, which can be expressed as $E_{i}=\{e_{i}^{s_{1}},\;e_{i}^{s_{2}},\ldots ,e_{i}^{s_{m}}\}$. Accordingly, a larger abundance vector containing all the sncRNA species within the same family across all the samples can be expressed as $E=\{e_{1}^{s_{1}},\;e_{1}^{s_{2}},\ldots ,e_{1}^{s_{m}},\ldots ,e_{n}^{s_{1}},\;e_{n}^{s_{2}},\ldots ,e_{n}^{s_{m}}\}$. A linear model comparing the overall abundance of the sncRNA family between two groups can be constructed as $E={\beta_{0}+\beta }_{1}G+\beta_{2}{\mathrm{RNA}}_{2}+\beta_{3}{\mathrm{RNA}}_{3}+\cdots +\beta_{n}{\mathrm{RNA}}_{n}$, where *G* is a binary vector containing the group information or a numeric vector of continuous phenotype for each data point in *E*, while *RNA_i_* is a binary vector defining sncRNA *i* for each data point in *E*. The overall differential abundance of the given sncRNA family can be evaluated by the *P*-value and *t*-statistic of the variable *G*.

### 2.2 *FUSION_ps* module

The *FUSION_ps* module is designed to identify differentially abundant sncRNA families between paired samples (e.g. paired normal and tumor tissues) or to prioritize dysregulated sncRNA families for pilot studies with limited sample size, i.e. 1-on-1 comparison. We assume, for a given sncRNA family, there are *n* sncRNA species derived from this family and the direction of dysregulation is consistent across all these sncRNA species. For each sncRNA species *i* that is derived from this given sncRNA family, we collect the reads per million (RPM) values of each of the paired samples. The abundance of sncRNA species *i* in sample 1 (*s*_1_) and sample 2 (*s*_2_) can be expressed as $e_{i}^{s_{1}}$ and $e_{i}^{s_{2}}$, respectively. Accordingly, the abundance vectors of the *n* sncRNA species for *s*_1_ and *s*_2_ can be expressed as $E_{s_{1}}=\{e_{1}^{s_{1}},\;e_{2}^{s_{1}},\ldots ,e_{n}^{s_{1}}\}$ and $E_{s_{2}}=\{e_{1}^{s_{2}},\;e_{2}^{s_{2}},\ldots ,\;e_{n}^{s_{2}}\}$, respectively. *Wilcoxon* signed-rank test (also known as the paired-sample *Wilcoxon* test) can be used to compare the paired profiles between $E_{s_{1}}$ and $E_{s_{2}}$, which is restricted to the sncRNA species derived from the same family. The differential abundance of the given sncRNA family between the paired samples (i.e. *s*_1_ and *s*_2_) can be evaluated by the *P*-value. The positive-rank sum (*W^+^*) and negative-rank sum (*W*^−^), can be used to determine the direction of dysregulation between the two samples. If *W^+^* > *W*^−^, the given sncRNA family is upregulated in *s_1_* in comparison with *s*_2_, and *vice versa*. To calculate *W^+^* and *W*^−^, we first compute the absolute values of the difference ($D=\{{|d}_{1}|,|d_{2}|,\ldots |d_{n}|\},\;d_{i}=e_{i}^{s_{1}}-e_{i}^{s_{2}}$) between $E_{s_{1}}$ and $E_{s_{2}}$. The sncRNA species within the same family are further ranked based on the vector *D* and can be expressed as $R=\{r_{1},r_{2},\ldots ,r_{n}\}$. Accordingly, *W^+^* and *W^−^* can be calculated as follows:


$$W^{+}=\sum_{i\in [1,n],{\;e}_{i}^{s_{1}}>e_{i}^{s_{2}}}r_{i}$$

$$W^{-}=\sum_{i\in [1,n],{\;e}_{i}^{s_{1}}<e_{i}^{s_{2}}}r_{i}$$

### 2.3 Publicly available sncRNA-seq data

The performance of *FUSION* was tested using publicly available sncRNA-seq data. For the *FUSION_ms* module, two independent datasets were obtained from the Gene Expression Omnibus (GEO) database, which were generated by sncRNA-seq in human plasma from controls and patients with pancreatic ductal adenocarcinoma (PDAC), with one from the United States (USA) and the other one from Canada (CAN). The USA cohort contains six controls and five PDAC patients, and the libraries were constructed using QIAseq miRNA Library kit (GEO accession: GSE226762) (Liu *et al.* 2023b). The CAN cohort is composed of 13 controls and 16 PDAC patients, and the libraries were constructed using the CleanTag Small RNA Library Prep kit (GEO accession: GSE221185) (Roy *et al.* 2023). For the *FUSION_ps* module, we also obtained two independent datasets from the GEO database. These two datasets were generated by sncRNA-seq in paired normal and tumor tissues from patients with lung adenocarcinoma (LUAD), with one from China (CHN) and the other from South Korea (KOR). The CHN cohort contains 19 normal-tumor pairs without detailed information on library preparation (GEO accession: GSE244311) (Guo *et al.* 2024). The KOR cohort is composed of 48 normal-tumor pairs, and the libraries were constructed using the Illumina Small RNA Prep kit (GEO accession: GSE110907) (Yu *et al.* 2019).

### 2.4 Preprocess of the sncRNA-seq data

The raw reads of the sncRNA-seq data were first adapter-trimmed using *Cutadapt* (v4.5) (Martin 2011). The annotation and quantification of sncRNAs were performed using the *SPORTS* tool (v1.1) (Shi *et al.* 2018), with a maximum mismatch score of one and all other parameters set to default settings. RPM was used to measure the abundance of the individual sncRNA. For downstream comparative analysis, sncRNA species with a mean RPM > 0.1 across all the samples in each study cohort were included.

### 2.5 Differential abundance analysis using *FUSION*

The noncanonical sncRNA families considered in this study are listed in Table S1, available as supplementary data at *Bioinformatics* online, including tsRNAs, rsRNAs, and ysRNAs. Firstly, the *FUSION*_ms module was employed to identify the differentially abundant sncRNA families in the USA and CAN cohorts using default settings. Only the sncRNA families with at least two sncRNA species were retained. If the number of sncRNA species in a given sncRNA family exceeded 1000, only the top 1000 sncRNA species (ranked by *RPM*) will be considered for further analysis. For comparison with *FUSION_ms*, the conventional strategy using *t*-test was also applied to identify the differentially abundant sncRNA families, which was based on the summed-up *RPM* of the individual sncRNA species from each sncRNA family. Secondly, the *FUSION*_ps module was applied to identify the differentially abundant sncRNA families for each sample pair in the CHN and KOR cohorts using default settings. *P*-values were adjusted for multiple testing using *Bonferroni* correction for both the *FUSION_ms* and *FUSION_ps* analyses as well as the conventional *t*-test. The sncRNA families with adjusted *P *< .05 were deemed differentially abundant. The runtime of *FUSION* on each dataset is listed in Table S2, available as supplementary data at *Bioinformatics* online.

### 2.6 Differential abundance analysis using *DESeq2*

For comparative analysis with *FUSION*, *DESeq2* (v1.42.1) (Love *et al.* 2014) was employed using default settings for differential abundance analysis of sncRNA species and sncRNA families, which included normalization of raw read counts using the median-of-ratios method, dispersion estimation using empirical Bayes shrinkage, and differential abundance assessment using a negative binomial model. The read counts of sncRNA species and combined read counts of sncRNA species from individual sncRNA families were used as input for the differential abundance analysis of sncRNA species and sncRNA families, respectively.

### 2.7 Visualization of tRNA secondary structure

The information on tRNA secondary structure was obtained from GtRNAdb (Chan and Lowe 2009, Chan and Lowe 2016). The visualization of tRNA secondary structure was performed using the *forna* tool (Kerpedjiev *et al.* 2015).

## 3 Results

### 3.1 *FUSION_ms* outperforms conventional methods in identifying common dysregulated sncRNA families across independent cohorts

**Figure 1.**
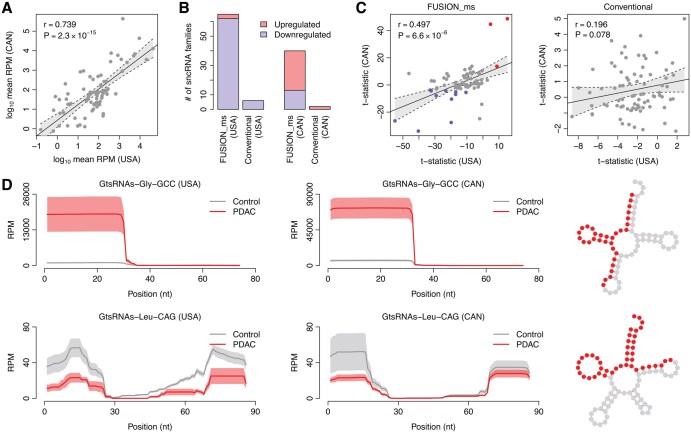
Dysregulated noncanonical sncRNA families unveiled by *FUSION_ms* in the USA and CAN cohorts. (A) Correlation in abundance of noncanonical sncRNA families between the USA and CAN cohorts. Each dot represents one sncRNA family. The abundance of each sncRNA family was measured as the *log*_10_-transformed mean *RPM* across all the samples. The correlation coefficient (*r*) and *P*-value were computed by the *Pearson* correlation test. (B) Number of differentially abundant sncRNA families prioritized by both *FUSION_ms* and conventional approaches. (C) Correlation in the *t*-statistic between the two cohorts. The *t*-statistic was computed by *FUSION_ms* and *t*-test. Each dot represents one sncRNA family. Red dots stand for the commonly upregulated sncRNA families, while blue ones denote the commonly downregulated sncRNA families in the two cohorts. The correlation coefficient (*r*) and *P*-value were computed by the *Pearson* correlation test. (D) Coverage pattern of two tsRNA families. GtsRNA-Gly-GCC is mainly derived from the 5′ end of the parental tRNA, while GtsRNA-Leu-CAG is derived from both ends of the parental tRNA. The solid curves indicate the mean *RPM* along the parental tRNAs, and the bands represent the standard error of the mean. The red nucleotides within the tRNA secondary structures highlight the location from which the tsRNAs are mainly derived.

The *FUSION_ms* module is optimized for multiple-sample comparison to detect family-level sncRNA abundance changes. To evaluate the effectiveness of *FUSION_ms*, we compared its performance against a conventional method for detecting differentially abundant sncRNA families. Our analysis focused on two objectives: (i) assessing the number of significantly dysregulated sncRNA families within individual cohorts and (ii) determining the ability to detect common sncRNA changes across independent cohorts studying the same disease. For this comparison, we analyzed two sncRNA-seq datasets of plasma samples from both controls and patients with PDAC, i.e. the USA and CAN cohorts, which were generated by distinct research teams (datasets sourced from the GEO database). We first confirmed the comparability of the two cohorts by examining the abundance of noncanonical sncRNA families. A strong positive correlation was observed between the USA and CAN cohorts (Fig. 1A), indicating similar sncRNA landscapes and providing a solid basis for further comparison in methodology.

Using *FUSION_ms*, we identified 65 and 40 differentially abundant sncRNA families between the control and PDAC samples in the USA and CAN cohort, respectively (Fig. 1B). In contrast, the conventional method, which measured the abundance of a given sncRNA family by summing up the *RPM* of individual sncRNA species within this sncRNA family, detected only six and two differentially abundant sncRNA families (*t*-test: adjusted *P *< .05) in the USA and CAN cohorts, respectively (Fig. 1B). This marked difference underscores the greater sensitivity of *FUSION_ms* in identifying disease-associated sncRNA alterations.

Next, we assessed whether *FUSION_ms* and the conventional method could identify sncRNA families consistently dysregulated across the two independent cohorts. A significant positive correlation was observed in *t*-statistic computed by *FUSION_ms* between the USA and CAN cohorts along with 14 sncRNA families showing consistent dysregulation in both cohorts (Fig. 1C and Table S3, available as supplementary data at *Bioinformatics* online), which suggests that *FUSION_ms* largely captured the common signal of PDAC-induced sncRNA alteration shared by these cohorts. Conversely, the conventional method showed no significant correlation in *t*-statistics and failed to identify any commonly dysregulated sncRNA families between the two cohorts (Fig. 1C).

Figure 1D illustrates the mapping patterns of two exemplary tsRNA families on their parental tRNAs: GtsRNA-Gly-GCC, which was commonly upregulated, and GtsRNA-Leu-CAG, which was commonly downregulated in the PDAC group across both cohorts. The coverage patterns visually confirm the differential abundance of these tsRNA families, which was successfully detected by *FUSION_ms*. In contrast, the conventional approach failed to identify these consistent changes across the datasets (Table S3, available as supplementary data at *Bioinformatics* online).

To further evaluate the performance of *FUSION_ms*, we applied *DESeq2* (Love *et al*. 2014), a widely used computational tool for differential expression analysis, to identify the dysregulated noncanonical sncRNA families at both family- and species-level data. The *Wald*-statistic computed by *DESeq2* was used as a proxy of the direction and degree of differential abundance. We found that there was no significant correlation in *Wald*-statistic at sncRNA family-level between the USA and CAN cohorts, while only a very weak correlation in *Wald*-statistic was observed at sncRNA species-level between the two cohorts (Fig. S1, available as supplementary data at *Bioinformatics* online), which indicates that *DESeq2*, like the conventional approach using *t*-test, could not detect consistent sncRNA changes across independent cohorts. Additionally, we also investigated whether *FUSION_ms*, the conventional approach using *t*-test, and *DESeq2* could identify miRNA families consistently dysregulated across the two independent cohorts. A statistically significant but relatively weak positive correlation was observed in *t*-statistic computed by *FUSION_ms* between the two cohorts along with 34 miRNA families showing consistent dysregulation in both cohorts (Fig. S2, available as supplementary data at *Bioinformatics* online). In contrast, *t*-test and *DESeq2* failed to identify any commonly dysregulated miRNA families, and no correlation in *t*-statistic (*t*-test) and in *Wald*-statistic (*DESeq2*) was observed between the two cohorts (Fig. S2, available as supplementary data at *Bioinformatics* online). All these results suggest that *FUSION_ms* not only substantially increases the statistical power of sncRNA family-level differential abundance analysis but also excels at uncovering consistent disease-related sncRNA changes across independent studies, a capability where conventional methods fall short.

**Figure 2.**
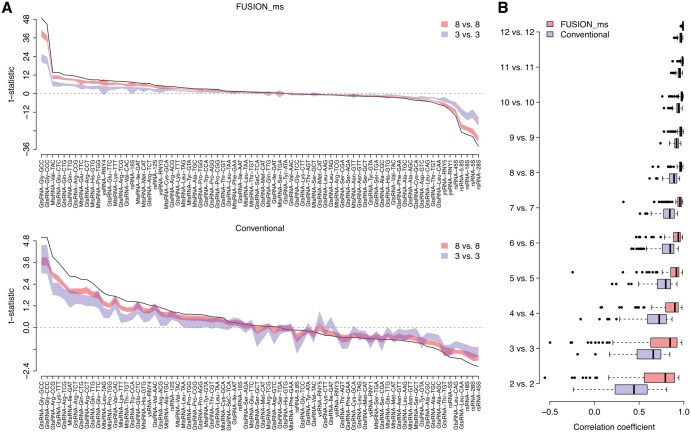
Robustness of *FUSION_ms* to sample size reduction in the CAN cohort. (A) Comparison of *t*-statistics. The black curves stand for the original *t*-statistics computed by *FUSION_ms* (upper panel) and the conventional approach (lower panel) without sample size reduction (i.e. 13 controls versus 16 patients). The color bands represent the 95% confidence interval in *t*-statistics of the resampling test with reduced sample sizes: (i) eight controls versus eight patients (red band) and (ii) three controls versus three patients (blue band). (B) Similarity between the original and resampled *t*-statistics. The *Pearson* correlation test was applied to measure the similarity. A more positive correlation coefficient implies higher similarity between the original *t*-statistic and the ones generated by resampling tests.

### 3.2 *FUSION_ms* maintains reliability with reduced sample sizes

We further tested whether *FUSION_ms* remains effective when fewer samples are available, a situation common in studies with limited data. To do this, we used a resampling test, which involves repeatedly analyzing smaller, randomly selected samples from our original datasets—the USA cohort (six controls and five patients) and the CAN cohort (13 controls and 16 patients). Our goal was to compare *FUSION_ms* against the conventional strategy and see how well each handles smaller sample sizes. From each cohort, we randomly picked *n* samples from the control group and *n* samples from the PDAC (patient) group. Differential abundance analysis was performed between the *n* random controls and *n* random patients (i.e. *n* versus *n* comparison), using *FUSION_ms* and conventional strategy, respectively. The *t*-statistics computed by both approaches were recorded. We repeated this procedure for 100 rounds for each *n* (*n *= 2, 3, 4 for the USA cohort and *n *= 2, 3, …, 12 for the CAN cohort). This resampling test revealed distinct robustness to sample size reduction between the two methods. The *t*-statistics computed by *FUSION_ms* exhibited a consistent landscape with reduced sample sizes as illustrated in Fig. 2A and Fig. S3A, available as supplementary data at *Bioinformatics* online. In contrast, the conventional approach displayed notable variability in *t*-statistics as the sample size decreased, suggesting a decline in reliability under such conditions. We further investigated the similarity between the *t*-statistics derived from the original dataset and those calculated from the resampled datasets using a correlation test. We found that, for each resampled *n* versus *n* comparison, the correlation coefficient is significantly higher (*Wilcoxon* rank sum test: *P *< 10^−8^) for *FUSION_ms* compared to the conventional method (Fig. 2B and Fig. S3B, available as supplementary data at *Bioinformatics* online). This observed stability of *FUSION_ms* against sample size variation highlights its robustness in sncRNA differential abundance analysis and distinguishes it from the conventional approaches that show greater sensitivity to sample size reductions, which suggests that *FUSION_ms* offers a reliable framework for sncRNA-seq studies where large cohorts are challenging to assemble.

**Figure 3.**
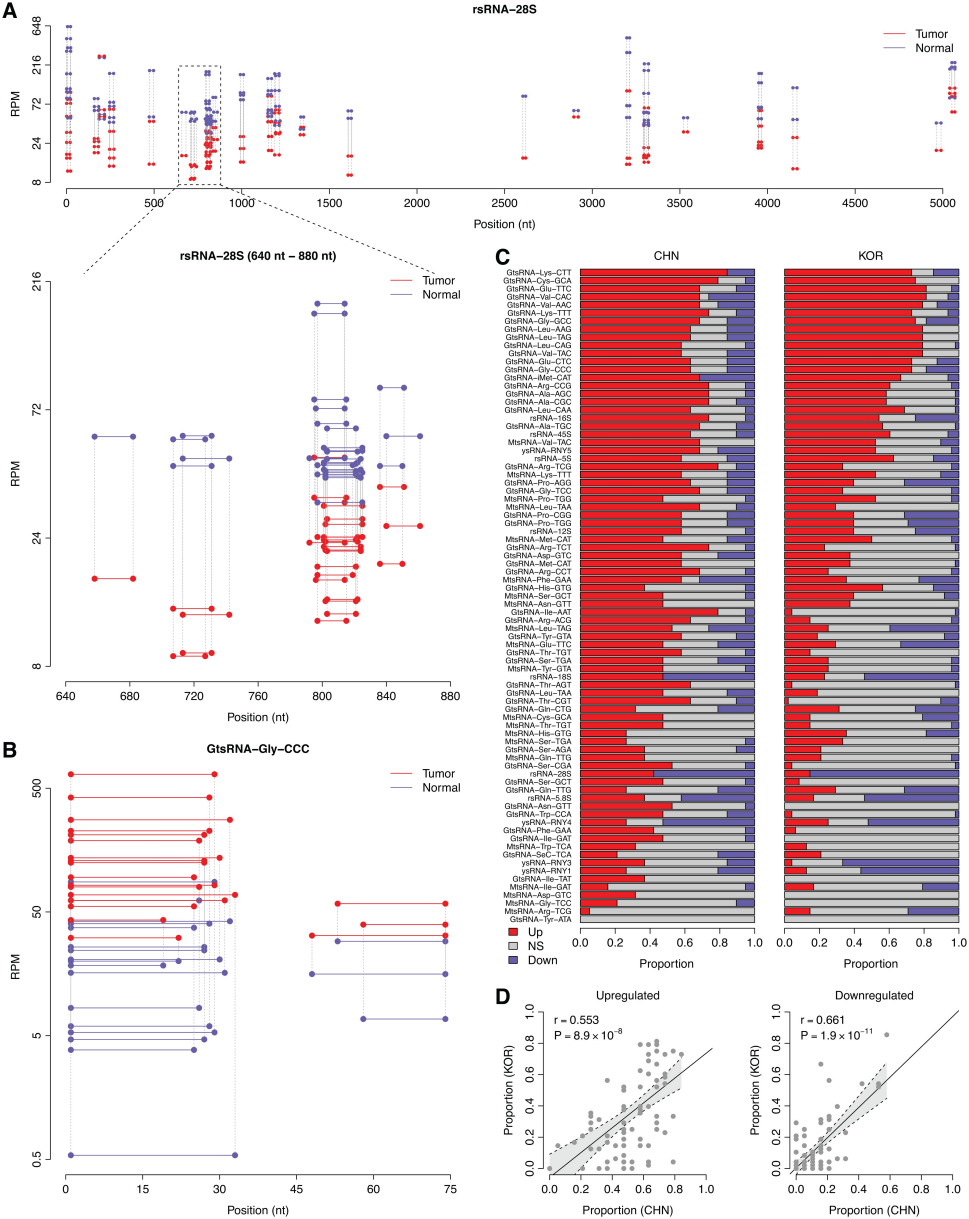
Identification of personal dysregulated noncanonical sncRNA families in LUAD. (A) Exemplary paired comparison of rsRNA-28S for a LUAD patient from the CHN cohort. The rsRNA-28S species were mapped to the parental rRNA individually. Only the top 100 rsRNA species (ranked by mean *RPM*) are demonstrated in the upper panel. Each horizontal line denotes a single rsRNA species. The vertical dash lines highlight the paired rsRNA species from the normal (blue) and tumor (red) tissues, respectively. The lower panel provides a zoom-in view of the rRNA region from 640 to 880 nt. (B) Exemplary paired comparison of GtsRNA-Gly-CCC for a LUAD patient from the CHN cohort. The GtsRNA-Gly-CCC species were mapped to the parental tRNA individually. Only the top 20 tsRNA species (ranked by mean *RPM*) are demonstrated here. (C) Proportion of LUAD patients exhibiting sncRNA family-level dysregulation in tumor tissue. Red represents upregulation; blue denotes downregulation; gray stands for non-significant (NS). (D) Correlation in proportion of patients with sncRNA family-level dysregulation (relative to normal tissue) between the CHN and KOR cohorts. Each dot represents one sncRNA family. The correlation coefficient (*r*) and *P*-value were computed by the *Pearson* correlation test.

### 3.3 *FUSION_ps* identifies personal dysregulated sncRNA families in LUAD

The *FUSION_ps* module is tailored for paired-sample comparisons, enabling “1-on-1” differential abundance analysis of sncRNA families in single-case studies. Traditional methods often falter in such scenarios due to limited statistical power, particularly when sncRNA species are aggregated into individual categories (e.g. summing up all reads for GtsRNA-Gly-CCC into one value). In contrast, *FUSION_ps* processes sncRNAs by first quantifying individual species and then grouping them into families, such as GtsRNA-Gly-CCC. This approach retains detailed sncRNA abundance information, ensuring the statistical power in single-case comparison. To assess the performance of *FUSION_ps*, we analyzed two independent LUAD sncRNA-seq datasets obtained from the GEO database: the CHN (containing 19 normal-tumor pairs) and KOR (containing 48 normal-tumor pairs) cohorts. Figure 3A demonstrates an exemplary paired comparison for an LUAD patient from the CHN cohort, indicating a clear downregulation of the rsRNA-28S family in tumor tissue compared to normal tissue, while Figure 3B and Fig. S4, available as supplementary data at *Bioinformatics* online demonstrate an exemplary paired comparison for a LUAD patient from the CHN cohort, revealing a marked upregulation of the GtsRNA-Gly-CCC family in tumor tissue compared to normal tissue. As individual patients may have different disease mechanisms, we expanded this analysis to all the patients in both cohorts and explored the personal sncRNA dysregulation patterns using *FUSION_ps* (Fig. 3C). For example, in the CHN cohort, 63.2% of patients exhibited upregulation of GtsRNA-Gly-CCC in tumor tissue, while 15.8% showed downregulation; in the KOR cohort, these figures were 72.9% and 18.8%, respectively (Fig. 3C). Remarkably, we observed a significant positive correlation between the two cohorts in the proportion of patients with upregulation of specific sncRNA families (Fig. 3D). This trend was similarly reflected in the proportion of patients with downregulation (Fig. 3D). These findings demonstrated that *FUSION_ps* not only detects personal sncRNA changes within individual patients but also identifies consistent dysregulation patterns across independent studies. This capability highlights the reliability of the *FUSION_ps* module and its potential to reveal insights that might be obscured in traditional group-based analysis.

In addition to the analysis on noncanonical sncRNAs, we also tested the performance of *FUSION_ps* on miRNAs by assessing whether *FUSION_ps* can detect similar dysregulation patterns in miRNA families between the CHN and KOR cohorts. As a result, *FUSION_ps* revealed a similar proportion of LUAD patients exhibiting miRNA family-level up-/downregulation in tumor tissue (Fig. S5, available as supplementary data at *Bioinformatics* online), which further suggests the robustness of *FUSION_ps* in identifying consistent sncRNA changes between independent cohorts.

## 4 Discussion

The sncRNA research has evolved significantly, shifting from the well-characterized miRNAs to the emerging classes such as tsRNAs, rsRNAs, and ysRNAs (Shi *et al.* 2022, Chen and Zhou 2023). These molecules, shaped by intricate biogenesis pathways involving RNases and RNA modification enzymes (Chen *et al.* 2021), could either exhibit RNAi-like function on their target RNAs (Kuscu *et al.* 2018, Guan *et al.* 2020, Guan and Grigoriev 2021) or go beyond linear sequence complementarity, such as aptamer-like mechanisms relying on secondary and tertiary RNA structures (Chen and Zhou 2023, Yu *et al.* 2025). This complexity challenges traditional methods for annotating and quantifying sncRNAs, which struggle to balance resolution, noise, and biological relevance. To overcome these limitations, we previously developed the *SPORTS* tool for precise sncRNA identification and annotation (Shi *et al.* 2018). Building on this, our new tool, *FUSION*, enhances quantitative analysis by integrating individual sncRNA species with their parental RNA families, enabling robust differential abundance studies even with limited sample sizes.

*FUSION* was designed to overcome challenges in traditional sncRNA data processing. Conventional methods either sum all sequencing reads mapped to a parental RNA, sacrificing the resolution of individual sncRNA species, or treat each unique RNA sequence independently, introducing noise in low-replicate settings and diminishing the biological relevance tied to the origin of each sncRNA. *FUSION* bridges these approaches by first quantifying unique sncRNA species and then aggregating them into their respective parental RNA families. This method captures the contributions of individual species while improving statistical power and robustness for differential abundance analysis. The approach is analogous to pathway analyses on differentially expressed genes, where grouping pathway-related genes potentially enhances biological insights compared to analyzing individual genes alone, particularly with smaller sample sizes or in a single-case study (Gardeux *et al.* 2014). *FUSION* includes two modules: *FUSION_ms*, for analyzing sncRNA datasets even with limited samples (e.g. publicly available in the GEO database) and *FUSION_ps*, for paired comparison in personalized medicine. Together, these tools address the growing demands of sncRNA research across diverse applications.

In biomedical research, larger sample sizes typically enhance statistical accuracy but can be impractical or excessive for studies with limited resources, while very small sample sizes often produce unreliable results (Andrade 2020). The two most widely used tools for RNA differential expression analysis, *DESeq2* and *edgeR*, are optimized for sample sizes of up to eight per condition (Li *et al.* 2022). However, these tools are not well-suited for sncRNA analysis due to the challenges such as high sequence heterogeneity and noise when analyzing individual sncRNA sequences, as well as the loss of critical sequence-specific information when sncRNAs are grouped into family-level categories. Our *FUSION* tool addresses these limitations by offering a robust solution for sncRNA differential abundance analysis, particularly with limited sample sizes. Benchmarking tests show that *FUSION* outperforms conventional approaches like *DESeq2*, delivering greater statistical power and consistency even when sample sizes are reduced. For example, *FUSION* consistently identified sncRNA changes across independent studies from different labs, where conventional methods could not. This makes *FUSION* a significant advancement for sncRNA research, particularly in resource-constrained settings.

Beyond its robustness with limited samples, *FUSION* also supports precision medicine through the *FUSION_ps* module. *FUSION_ps* excels in paired-sample comparisons, enabling “1-on-1” differential abundance analysis of sncRNA families in single-case studies. This feature is particularly valuable for personalized clinical care, where understanding individual responses is increasingly critical. Unlike traditional methods, which struggle with 1 versus 1 comparison due to limited statistical power, *FUSION_ps* offers a robust solution. For instance, it can track changes in an individual’s sncRNA profiles before and after treatment, providing a biologically relevant measure of therapeutic impact.

While *FUSION* preserves sensitivity by capturing all relevant sncRNA species, including tsRNAs, it is important to note that many tRNAs—particularly those in conserved regions from which tsRNAs often originate—are highly similar or even identical. By default, *FUSION* includes all sncRNA species that map to a given parent RNA, thereby preserving sensitivity but potentially leading to a higher false-positive rate. An alternative strategy is to focus on sncRNA species with unique parental RNA annotations. In such cases, filtering only for uniquely mapped reads would exclude a substantial fraction of biologically meaningful sncRNAs, increasing the risk of false negatives more than false positives. However, if a user prioritizes focusing on sncRNAs with unique parental origin and is more concerned about potential false positives arising from multi-mapping sncRNAs, *FUSION* provides an option to restrict the analysis to sncRNA species with unique parental RNA annotations. Actually, a strong positive correlation was observed between the results generated using the two different settings (Figs S6 and S7, available as supplementary data at *Bioinformatics* online). However, some dots deviated from the diagonal, which potentially arises from the sncRNA families consisting of a considerable proportion of non-uniquely mapped sequences. Restricting *FUSION* analysis to uniquely annotated reads may pose a marked effect on statistical power for these sncRNA families, which explains the observed deviations.

Recent efforts in sncRNA differential abundance analysis have targeted individual sequences, families, or both, with a focus on tsRNAs. Tools like *tRAX* (Holmes *et al.* 2022) and *tReasure* (Lee *et al.* 2022) represent these advancements. However, *tRAX* either combines read counts from tsRNAs and other small RNAs, reducing resolution, or analyzes individual sncRNA sequences, introducing noise—issues reminiscent of conventional approaches. Similarly, *tReasure* relies on combined read counts of mapped sncRNAs, often losing the information regarding individual sequence versatility. In contrast, *FUSION* outperforms these tools by capturing individual sncRNA sequence information and integrating it into family-level analysis without sacrificing biological meaning. With these advantages, we believe *FUSION* not only enhances sncRNA data analysis but also paves the way for precision diagnostics and therapeutic innovation.

In future applications, *FUSION*’s reports on altered sncRNA abundance can guide functional studies of these sncRNAs. For example, one can test the RNAi-like targeting potential of tsRNAs/rsRNAs by leveraging CLASH experiments, which directly capture sncRNA–target interactions through sequencing of chimeric RNA hybrids (Guan *et al.* 2020, Guan and Grigoriev 2021, Guan and Grigoriev 2023). In this way, *FUSION* can help prioritize biologically relevant candidates for validation in CLASH-based assays, rather than relying solely on computational predictions. Alternatively, functional investigations of sncRNAs may also explore beyond linear sequence complementarity, including aptamer-like mechanisms that depend on RNA structure and protein or metabolite binding (Chen and Zhou 2023, Yu *et al.* 2025). Meanwhile, we would like to emphasize that while *FUSION* predicts sncRNA family-level differential abundance, this does not imply that all fragments within a family are uniformly regulated or functionally equivalent. Individual rsRNAs/rRFs may deviate from the family trend due to localized biogenesis mechanisms and can exert distinct regulatory effects.

## Supplementary Material

btaf526_Supplementary_Data

## Data Availability

The source code of *FUSION*, along with example datasets, is available on GitHub (https://github.com/cozyrna/FUSION) and archived at https://doi.org/10.5281/zenodo.16929712. All the sncRNA-seq data analyzed in this study are publicly available in the GEO database (accession numbers: GSE226762, GSE221185, GSE244311, and GSE110907) as described in the Materials and methods section.
