# Supplementary material for: FUSION: a family-level integration approach for robust differential analysis of small non-coding RNAs
Source: Bioinformatics. 2025 Sep 18;41(10):btaf526. doi: 10.1093/bioinformatics/btaf526 (PMC12502913; doi:10.1093/bioinformatics/btaf526)
Supplement: btaf526_Supplementary_Data [file btaf526_supplementary_data.pdf]

Supplementary Material for Rawal *et al.*, "*FUSION*: a family-level integration approach for robust differential analysis of small noncoding RNAs"

Supplementary Table S1. Noncanonical sncRNA families

Category	Parental RNA template	Noncanonical sncRNA family
tsRNA	Genomic tRNA-Ala-AGC	Genomic tsRNA-Ala-AGC (GtsRNA-Ala-AGC)
tsRNA	Genomic tRNA-Ala-CGC	Genomic tsRNA-Ala-CGC (GtsRNA-Ala-CGC)
tsRNA	Genomic tRNA-Ala-TGC	Genomic tsRNA-Ala-TGC (GtsRNA-Ala-TGC)
tsRNA	Genomic tRNA-Arg-ACG	Genomic tsRNA-Arg-ACG (GtsRNA-Arg-ACG)
tsRNA	Genomic tRNA-Arg-CCG	Genomic tsRNA-Arg-CCG (GtsRNA-Arg-CCG)
tsRNA	Genomic tRNA-Arg-CCT	Genomic tsRNA-Arg-CCT (GtsRNA-Arg-CCT)
tsRNA	Genomic tRNA-Arg-TCG	Genomic tsRNA-Arg-TCG (GtsRNA-Arg-TCG)
tsRNA	Genomic tRNA-Arg-TCT	Genomic tsRNA-Arg-TCT (GtsRNA-Arg-TCT)
tsRNA	Genomic tRNA-Asn-GTT	Genomic tsRNA-Asn-GTT (GtsRNA-Asn-GTT)
tsRNA	Genomic tRNA-Asp-GTC	Genomic tsRNA-Asp-GTC (GtsRNA-Asp-GTC)
tsRNA	Genomic tRNA-Cys-GCA	Genomic tsRNA-Cys-GCA (GtsRNA-Cys-GCA)
tsRNA	Genomic tRNA-Gln-CTG	Genomic tsRNA-Gln-CTG (GtsRNA-Gln-CTG)
tsRNA	Genomic tRNA-Gln-TTG	Genomic tsRNA-Gln-TTG (GtsRNA-Gln-TTG)
tsRNA	Genomic tRNA-Glu-CTC	Genomic tsRNA-Glu-CTC (GtsRNA-Glu-CTC)
tsRNA	Genomic tRNA-Glu-TTC	Genomic tsRNA-Glu-TTC (GtsRNA-Glu-TTC)
tsRNA	Genomic tRNA-Gly-CCC	Genomic tsRNA-Gly-CCC (GtsRNA-Gly-CCC)
tsRNA	Genomic tRNA-Gly-GCC	Genomic tsRNA-Gly-GCC (GtsRNA-Gly-GCC)
tsRNA	Genomic tRNA-Gly-TCC	Genomic tsRNA-Gly-TCC (GtsRNA-Gly-TCC)
tsRNA	Genomic tRNA-His-GTG	Genomic tsRNA-His-GTG (GtsRNA-His-GTG)
tsRNA	Genomic tRNA-Ile-AAT	Genomic tsRNA-Ile-AAT (GtsRNA-Ile-AAT)
tsRNA	Genomic tRNA-Ile-GAT	Genomic tsRNA-Ile-GAT (GtsRNA-Ile-GAT)
tsRNA	Genomic tRNA-Ile-TAT	Genomic tsRNA-Ile-TAT (GtsRNA-Ile-TAT)
tsRNA	Genomic tRNA-iMet-CAT	Genomic tsRNA-iMet-CAT (GtsRNA-iMet-CAT)
tsRNA	Genomic tRNA-Leu-AAG	Genomic tsRNA-Leu-AAG (GtsRNA-Leu-AAG)
tsRNA	Genomic tRNA-Leu-CAA	Genomic tsRNA-Leu-CAA (GtsRNA-Leu-CAA)
tsRNA	Genomic tRNA-Leu-CAG	Genomic tsRNA-Leu-CAG (GtsRNA-Leu-CAG)
tsRNA	Genomic tRNA-Leu-TAA	Genomic tsRNA-Leu-TAA (GtsRNA-Leu-TAA)
tsRNA	Genomic tRNA-Leu-TAG	Genomic tsRNA-Leu-TAG (GtsRNA-Leu-TAG)
tsRNA	Genomic tRNA-Lys-CTT	Genomic tsRNA-Lys-CTT (GtsRNA-Lys-CTT)
tsRNA	Genomic tRNA-Lys-TTT	Genomic tsRNA-Lys-TTT (GtsRNA-Lys-TTT)
tsRNA	Genomic tRNA-Met-CAT	Genomic tsRNA-Met-CAT (GtsRNA-Met-CAT)
tsRNA	Genomic tRNA-Phe-GAA	Genomic tsRNA-Phe-GAA (GtsRNA-Phe-GAA)
tsRNA	Genomic tRNA-Pro-AGG	Genomic tsRNA-Pro-AGG (GtsRNA-Pro-AGG)
tsRNA	Genomic tRNA-Pro-CGG	Genomic tsRNA-Pro-CGG (GtsRNA-Pro-CGG)
tsRNA	Genomic tRNA-Pro-TGG	Genomic tsRNA-Pro-TGG (GtsRNA-Pro-TGG)
tsRNA	Genomic tRNA-SeC-TCA	Genomic tsRNA-SeC-TCA (GtsRNA-SeC-TCA)
tsRNA	Genomic tRNA-Ser-AGA	Genomic tsRNA-Ser-AGA (GtsRNA-Ser-AGA)
tsRNA	Genomic tRNA-Ser-CGA	Genomic tsRNA-Ser-CGA (GtsRNA-Ser-CGA)
tsRNA	Genomic tRNA-Ser-GCT	Genomic tsRNA-Ser-GCT (GtsRNA-Ser-GCT)
tsRNA	Genomic tRNA-Ser-TGA	Genomic tsRNA-Ser-TGA (GtsRNA-Ser-TGA)
tsRNA	Genomic tRNA-Thr-AGT	Genomic tsRNA-Thr-AGT (GtsRNA-Thr-AGT)
tsRNA	Genomic tRNA-Thr-CGT	Genomic tsRNA-Thr-CGT (GtsRNA-Thr-CGT)

Category	Parental RNA template	Noncanonical sncRNA family
tsRNA	Genomic tRNA-Thr-TGT	Genomic tsRNA-Thr-TGT (GtsRNA-Thr-TGT)
tsRNA	Genomic tRNA-Trp-CCA	Genomic tsRNA-Trp-CCA (GtsRNA-Trp-CCA)
tsRNA	Genomic tRNA-Tyr-ATA	Genomic tsRNA-Tyr-ATA (GtsRNA-Tyr-ATA)
tsRNA	Genomic tRNA-Tyr-GTA	Genomic tsRNA-Tyr-GTA (GtsRNA-Tyr-GTA)
tsRNA	Genomic tRNA-Val-AAC	Genomic tsRNA-Val-AAC (GtsRNA-Val-AAC)
tsRNA	Genomic tRNA-Val-CAC	Genomic tsRNA-Val-CAC (GtsRNA-Val-CAC)
tsRNA	Genomic tRNA-Val-TAC	Genomic tsRNA-Val-TAC (GtsRNA-Val-TAC)
tsRNA	Mitochondrial tRNA-Ala-TGC	Mitochondrial tsRNA-Ala-TGC (MtsRNA-Ala-TGC)
tsRNA	Mitochondrial tRNA-Arg-TCG	Mitochondrial tsRNA-Arg-TCG (MtsRNA-Arg-TCG)
tsRNA	Mitochondrial tRNA-Asn-GTT	Mitochondrial tsRNA-Asn-GTT (MtsRNA-Asn-GTT)
tsRNA	Mitochondrial tRNA-Asp-GTC	Mitochondrial tsRNA-Asp-GTC (MtsRNA-Asp-GTC)
tsRNA	Mitochondrial tRNA-Cys-GCA	Mitochondrial tsRNA-Cys-GCA (MtsRNA-Cys-GCA)
tsRNA	Mitochondrial tRNA-Gln-TTG	Mitochondrial tsRNA-Gln-TTG (MtsRNA-Gln-TTG)
tsRNA	Mitochondrial tRNA-Glu-TTC	Mitochondrial tsRNA-Glu-TTC (MtsRNA-Glu-TTC)
tsRNA	Mitochondrial tRNA-Gly-TCC	Mitochondrial tsRNA-Gly-TCC (MtsRNA-Gly-TCC)
tsRNA	Mitochondrial tRNA-His-GTG	Mitochondrial tsRNA-His-GTG (MtsRNA-His-GTG)
tsRNA	Mitochondrial tRNA-Ile-GAT	Mitochondrial tsRNA-Ile-GAT (MtsRNA-Ile-GAT)
tsRNA	Mitochondrial tRNA-Leu-TAA	Mitochondrial tsRNA-Leu-TAA (MtsRNA-Leu-TAA)
tsRNA	Mitochondrial tRNA-Leu-TAG	Mitochondrial tsRNA-Leu-TAG (MtsRNA-Leu-TAG)
tsRNA	Mitochondrial tRNA-Lys-TTT	Mitochondrial tsRNA-Lys-TTT (MtsRNA-Lys-TTT)
tsRNA	Mitochondrial tRNA-Met-CAT	Mitochondrial tsRNA-Met-CAT (MtsRNA-Met-CAT)
tsRNA	Mitochondrial tRNA-Phe-GAA	Mitochondrial tsRNA-Phe-GAA (MtsRNA-Phe-GAA)
tsRNA	Mitochondrial tRNA-Pro-TGG	Mitochondrial tsRNA-Pro-TGG (MtsRNA-Pro-TGG)
tsRNA	Mitochondrial tRNA-Ser-GCT	Mitochondrial tsRNA-Ser-GCT (MtsRNA-Ser-GCT)
tsRNA	Mitochondrial tRNA-Ser-TGA	Mitochondrial tsRNA-Ser-TGA (MtsRNA-Ser-TGA)
tsRNA	Mitochondrial tRNA-Thr-TGT	Mitochondrial tsRNA-Thr-TGT (MtsRNA-Thr-TGT)
tsRNA	Mitochondrial tRNA-Trp-TCA	Mitochondrial tsRNA-Trp-TCA (MtsRNA-Trp-TCA)
tsRNA	Mitochondrial tRNA-Tyr-GTA	Mitochondrial tsRNA-Tyr-GTA (MtsRNA-Tyr-GTA)
tsRNA	Mitochondrial tRNA-Val-TAC	Mitochondrial tsRNA-Val-TAC (MtsRNA-Val-TAC)
rsRNA	12S-rRNA	rsRNA-12S
rsRNA	16S-rRNA	rsRNA-16S
rsRNA	18S-rRNA	rsRNA-18S
rsRNA	28S-rRNA	rsRNA-28S
rsRNA	45S-rRNA	rsRNA-45S
rsRNA	5.8S-rRNA	rsRNA-5.8S
rsRNA	5S-rRNA	rsRNA-5S
ysRNA	RNY1-YRNA	ysRNA-RNY1
ysRNA	RNY3-YRNA	ysRNA-RNY3
ysRNA	RNY4-YRNA	ysRNA-RNY4
ysRNA	RNY5-YRNA	ysRNA-RNY5

Supplementary Table S2. Runtime of *FUSION* (at default parameters)

Dataset	Comparison type	Sample size	*FUSION* module	Runtime (seconds)*
GSE226762 (USA)	Multiple-sample comparison	6:5	*FUSION_ms*	52
GSE221185 (CAN)	Multiple-sample comparison	13:16	*FUSION_ms*	113
GSE244311 (CHN)	Paired-sample comparison	19:19	*FUSION_ps*	34
GSE110907 (KOR)	Paired-sample comparison	48:48	*FUSION_ps*	50

*All runs were performed on Intel® Xeon® w5-2445 Processor with 128GB RAM.

Supplementary Table S3. Commonly dysregulated noncanonical sncRNA families in the USA and CAN cohorts identified by *FUSION_ms*

sncRNA family	Direction of differential expression (relative to controls)	USA		CAN	
		t-statistic	Adjusted *P*	*t*-statistic	Adjusted *P*
GtsRNA-Ala-AGC	Downregulated	-32.95*	$< 10^{-16}$	-3.84	9.9×10^{-3}
GtsRNA-Asp-GTC	Downregulated	-32.35	$< 10^{-16}$	-4.38	9.7×10^{-4}
GtsRNA-Cys-GCA	Downregulated	-23.22*	$< 10^{-16}$	-4.35	1.1×10^{-3}
GtsRNA-Glu-CTC	Upregulated	8.72	2.6×10^{-16}	13.73	$< 10^{-16}$
GtsRNA-Gly-CCC	Upregulated	4.74	1.6×10^{-4}	44.73*	$< 10^{-16}$
GtsRNA-Gly-GCC	Upregulated	15.27	$< 10^{-16}$	48.80*	$< 10^{-16}$
GtsRNA-Leu-CAA	Downregulated	-17.05	$< 10^{-16}$	-6.96	3.7×10^{-10}
GtsRNA-Leu-CAG	Downregulated	-16.98	$< 10^{-16}$	-5.10	2.9×10^{-5}
GtsRNA-Thr-TGT	Downregulated	-10.60	$< 10^{-16}$	-5.51	3.6×10^{-6}
rsRNA-18S	Downregulated	-55.00	$< 10^{-16}$	-26.47	$< 10^{-16}$
rsRNA-28S	Downregulated	-40.35	$< 10^{-16}$	-34.04	$< 10^{-16}$
rsRNA-5S	Downregulated	-19.83	$< 10^{-16}$	-27.67	$< 10^{-16}$
ysRNA-RNY1	Downregulated	-19.61	$< 10^{-16}$	-10.42	$< 10^{-16}$
ysRNA-RNY5	Downregulated	-29.21	$< 10^{-16}$	-7.91	2.1×10^{-13}

* The sncRNA family exhibits an adjusted $P < 0.05$ if using the conventional method.

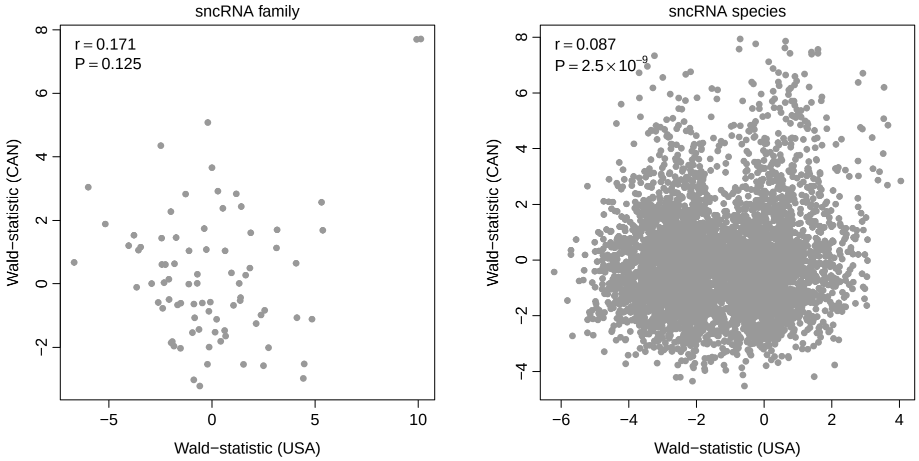

Supplementary Figure S1. Correlation in *Wald*-statistic of noncanonical sncRNA differential abundance analysis between the USA and CAN cohorts. The *Wald*-statistic was computed between the control and PDAC groups using the *DESeq2* tool at both sncRNA family- and species-level. Each dot represents one noncanonical sncRNA family (left panel) and one noncanonical sncRNA species (right panel). The correlation coefficient (r) and P-value were computed by *Pearson* correlation test.

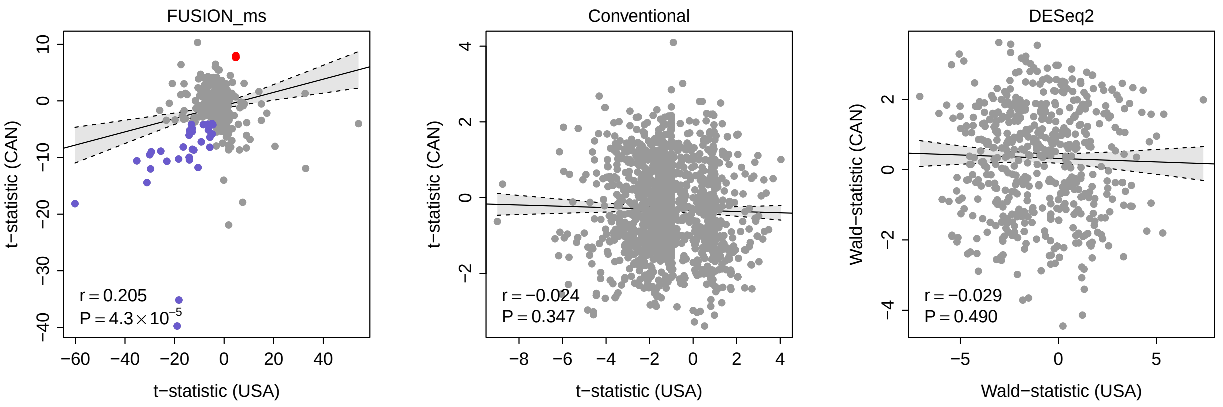

Supplementary Figure S2. Correlation in *t*-statistic and *Wald*-statistic of miRNA differential abundance analysis between the USA and CAN cohorts. The t-statistic in the left and middle panels was computed between the control and PDAC groups using *FUSION_ms* and t-test, respectively. The *Wald*-statistic in the right panel was computed using *DESeq2*. Each dot represents one miRNA family. Red dots stand for the commonly upregulated miRNA families while blue ones denote the commonly downregulated miRNA families in the two cohorts. The correlation coefficient (r) and P-value were computed by *Pearson* correlation test.

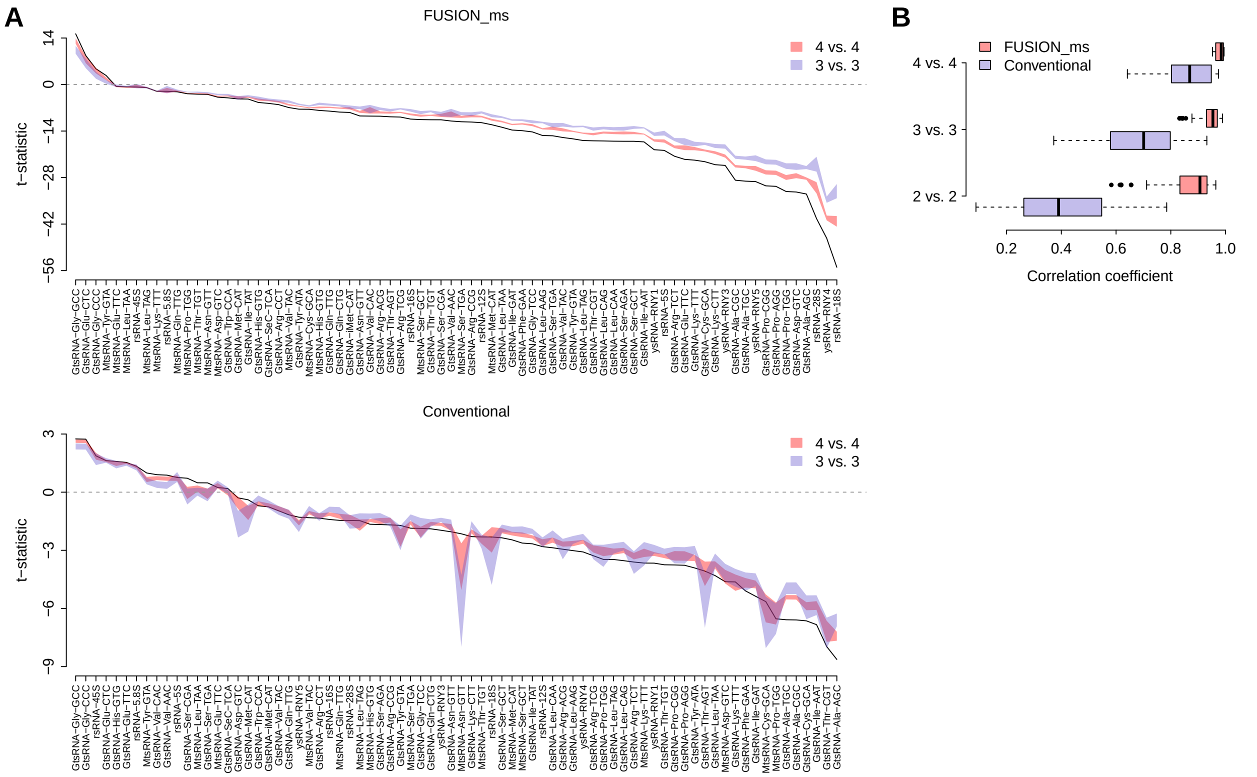

Supplementary Figure S3. Robustness of *FUSION_ms* to sample size reduction in the USA cohort. (A) Comparison of t-statistics. The black curves stand for the original t-statistics computed by *FUSION_ms* (upper panel) and the conventional approach (lower panel) without sample size reduction (*i.e.*, six controls *vs.* five patients). The color bands represent the 95% confidence interval in t-statistics of the resampling test with reduced sample sizes: i) four controls *vs.* four patients (red band) and ii) three controls *vs.* three patients (blue band). (B) Similarity between the original and resampled t-statistics. *Pearson* correlation test was applied to measure the similarity. More positive correlation coefficient implies higher similarity between the original t-statistic and the ones generated by resampling tests.

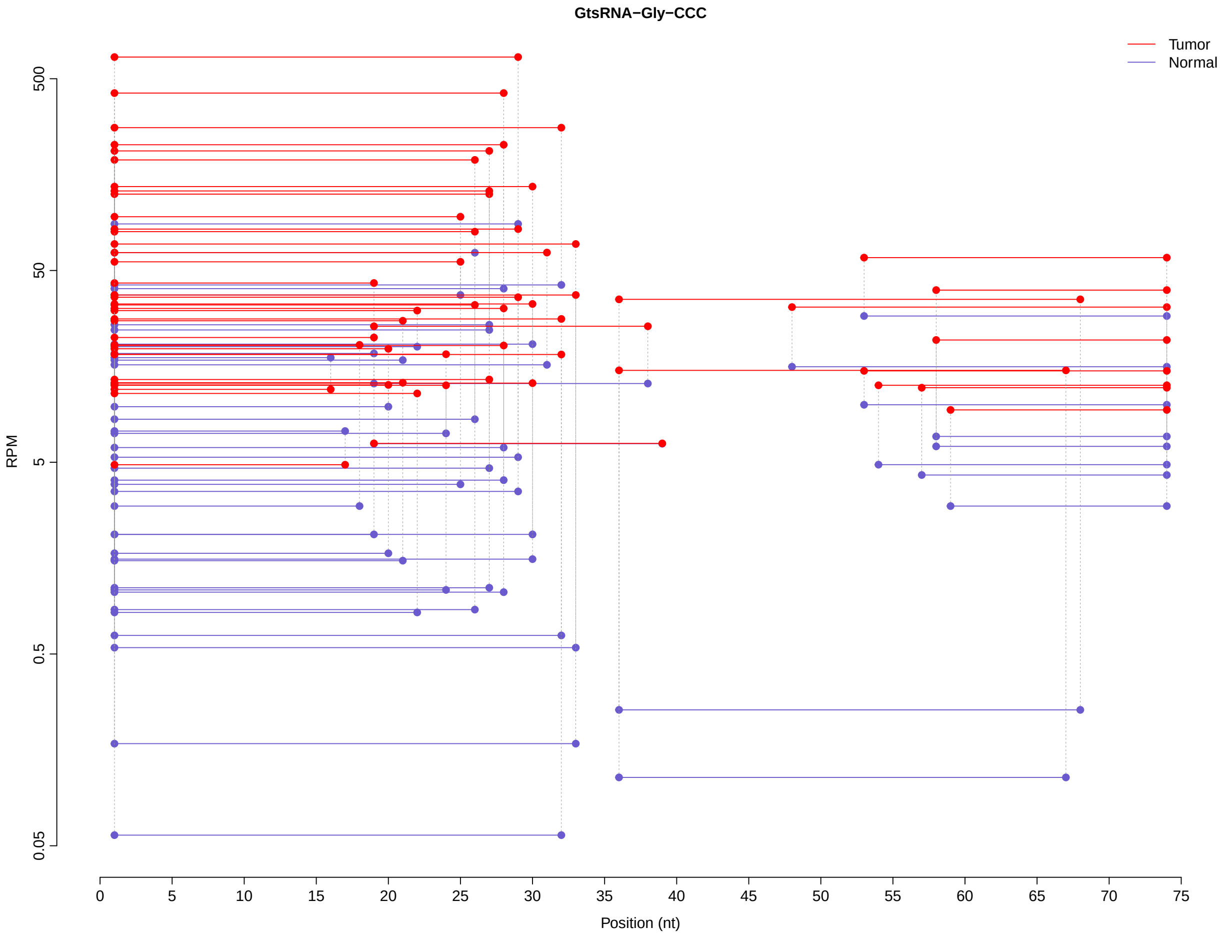

Supplementary Figure S4. Exemplary paired comparison of GtsRNA-Gly-CCC for a LUAD patient from the CHN cohort. The GtsRNA-Gly-CCC species were mapped to the parental tRNA individually. Only the top 50 tsRNA species (ranked by mean RPM) are demonstrated here. Each horizontal line denotes a single tsRNA species. The vertical dash lines highlight the paired tsRNA species from the normal (blue) and tumor (red) tissues, respectively.

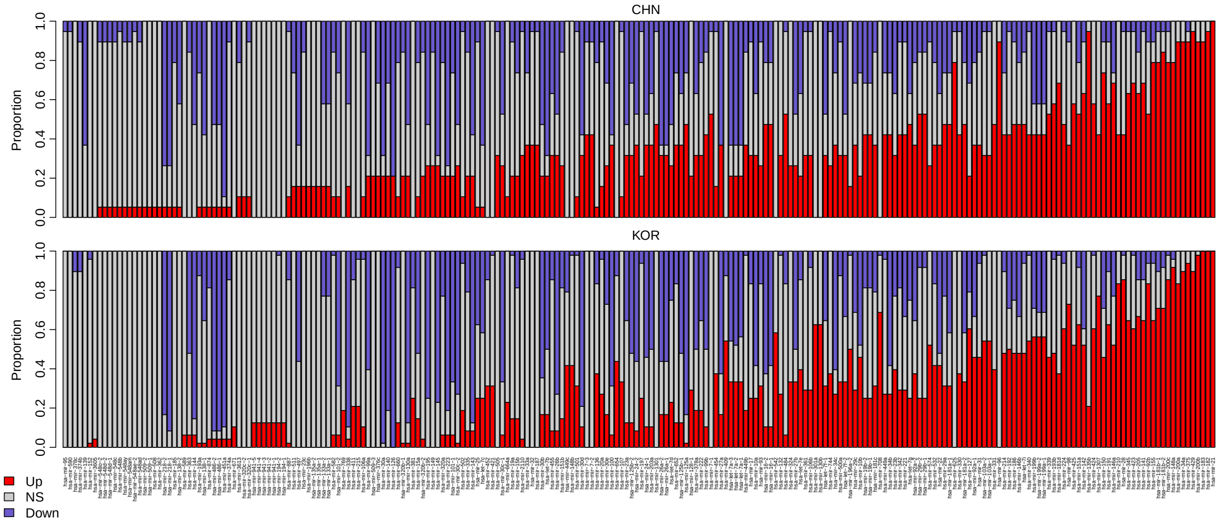

Supplementary Figure S5. Proportion of LUAD patients exhibiting miRNA family-level dysregulation in tumor tissue. Only the miRNA families with at least one normal-tumor pair exhibiting significant dysregulation were included. Red represents upregulation; blue denotes downregulation; grey stands for non-significant (NS).

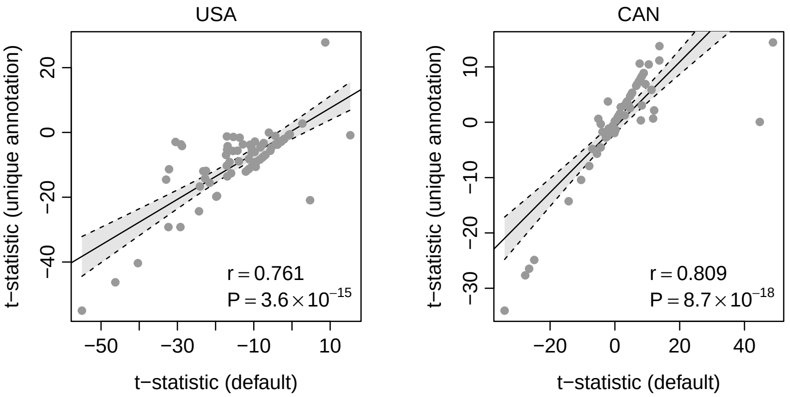

Supplementary Figure S6. Correlation between *FUSION_ms* mapping settings in the USA and CAN cohorts. The t-statistic generated using two different mapping settings was compared here. Each dot represents one noncanonical sncRNA family. X-axis: the t-statistic computed using the default setting considering all the sncRNA species that can be mapped to a parental RNA; Y-axis: the t-statistic computed based on the setting only considering the sncRNA species with unique parental RNA annotation. The correlation coefficient (r) and P-value were computed by *Pearson* correlation test.

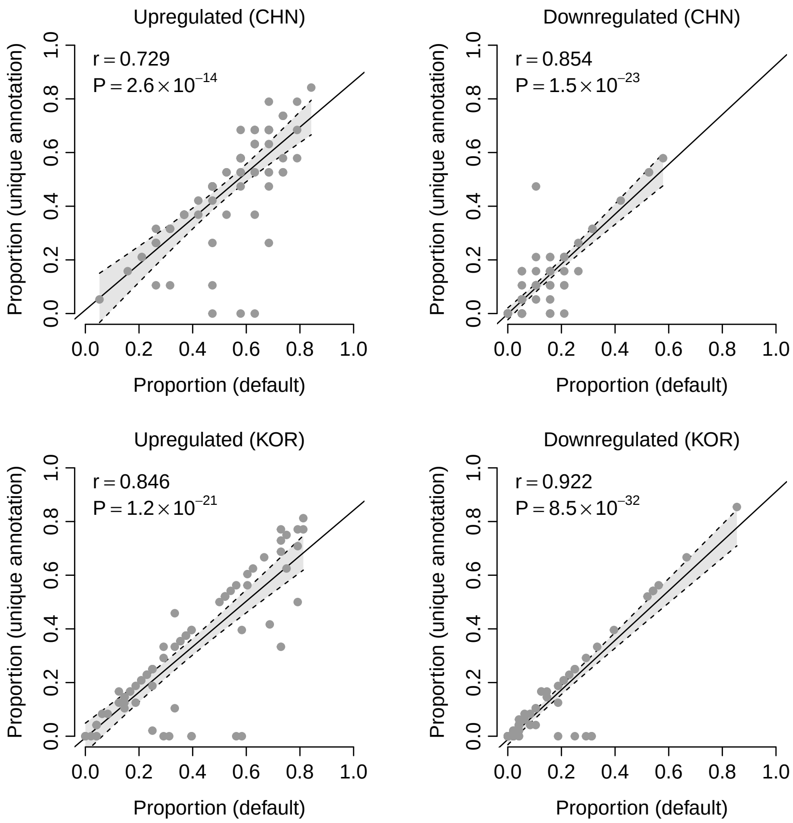

Supplementary Figure S7. Correlation between *FUSION_ps* mapping settings in the CHN and KOR cohorts. Two mapping settings were compared here. Each dot represents one noncanonical sncRNA family. X-axis: the proportion of LUAD patients exhibiting sncRNA family-level dysregulation in tumor tissue computed using the default setting considering all the sncRNA species that can be mapped to a parental RNA; Y-axis: the proportion of patients exhibiting sncRNA family-level dysregulation computed based on the setting only considering the sncRNA species with unique parental RNA annotation. The correlation coefficient (r) and P-value were computed by *Pearson* correlation test.
